# Hiking Trails Facilitate the Spread of a Native High‐Arctic Species

**DOI:** 10.1002/ece3.70809

**Published:** 2025-01-09

**Authors:** Deborah Zani, Heike Lischke, Jonas Åkerman, Veiko Lehsten

**Affiliations:** ^1^ Dynamic Macroecology/Land Change Science Swiss Federal Institute for Forest, Snow and Landscape Research WSL Birmensdorf Switzerland; ^2^ Department of Physical Geography and Ecosystem Science Lund University Lund Sweden; ^3^ Department of Natural Science, Design and Sustainable Development Mid Sweden University Östersund Sweden

**Keywords:** Arctic plants, dispersal model, *Papaver dahlianum*, species range shifts, tourism, trails, UAV

## Abstract

High‐Arctic environments are facing an elevated pace of warming and increasing human activities, making them more susceptible to the introduction and spread of alien species. We investigated the role of human disturbance in facilitating the spread of a native plant (*Papaver dahlianum*) in a high‐Arctic natural environment close to Isfjord Radio station and along adjacent hiking trails at Kapp Linné, Svalbard. We reconstructed the spatial pattern of the arrival and spread of *P. dahlianum* at Kapp Linné by combining historical records of the species occurrence (1928–2018) with a contemporary survey of the plant abundance along the main hiking trail (2023 survey) and tested the relative effects of altitude and proximity to hiking trails on the species density via a generalised linear model (GLM). We then compared historical records with the simulated annual spread of the species by assuming either only local spread or local spread plus spread from hiking trails. Finally, we used a fine‐scale UAV‐derived brightness index to test for terrain preference by applying a randomisation test. Distance from the station (56% explained variation) and minimum distance from the trail (28%) significantly explained the species density across the research area (best GLM *R* ^2^ = 0.755). The modelled species spread including the trail effect (fitted spread ~30 m yr.^−1^) managed to capture the maximum extent of the occupied area, whereas simulations assuming only local spread (~2 m yr.^−1^) underestimated the historical extent. A randomisation test showed that *P. dahlianum* has a significant preference for gravel soils with low vegetation cover due to either trail trampling and/or natural processes. Along with climate warming, human activities can increase the rate of species range shift by providing hot spots of introduction (human settlements) and spreading corridors (hiking trails). Our results show that spatially explicit models can be applied to accurately predict the potential spread of species, leading to a more efficient monitoring plan. Systematic monitoring of alien species and sanitisation measures should be prioritised in polar habitats with a high incidence of human disturbances.

## Graphical abstract

We reconstructed the arrival and spread of a native high‐Arctic plant species (*Papaver dahlianum*) in a high‐risk natural habitat of Svalbard by applying statistical analysis and spatial modelling on the species presence from historical records and a contemporary survey. Our results show that human activities can increase the rate of species range shift by providing hot spots of introduction (human settlements) and spreading corridors (hiking trails).

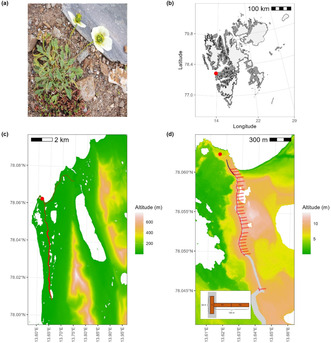

## 1 Introduction

Along with global climate change, human disturbance is considered one of the most important drivers of vegetation change in polar regions. High‐Arctic ecosystems have become vulnerable to extreme species redistribution due to polar amplification (Pearson et al. 2013; Serreze and Barry 2011), with temperatures rising at nearly the double rate of global averages (Box et al. 2019; Kattsov et al. 2005) and projected to further increase over the 21st century (Pörtner et al. 2019). Overall, more favourable (warmer) growing conditions can create new fundamental niches (suitable climates for establishment), thus allowing previously unfit species to survive in polar environments (Rew et al. 2020). This has led to the northward movement of many plant species worldwide, with an increasing threat from biological invasions of alien species (IAS) (i.e., species introduced by humans outside of their natural ranges) (Pecl et al. 2017; Wallingford et al. 2020). In addition, native plants may alter their spatial distribution to colonise newly suitable habitats (range shifters) (Rew et al. 2020; Urban 2020), as observed in the widely documented range expansion of native shrub species and the consequent decline in graminoid, bryophyte and lichen cover across the Arctic (Mekonnen et al. 2021). These changes in plant composition can cascade to other trophic levels, for example, by altering the availability of palatable forage for herbivores and further affecting herbivore–predator dynamics (Descamps et al. 2017; Hiltunen et al. 2022). More importantly, changes in the vegetation cover in polar regions and especially at large scales are expected to affect the surface albedo, along with potential carbon sequestration and greenhouse gas releases, thus potentially impacting warming trends at high latitudes (Pearson et al. 2013).

In combination with climate warming, human disturbances in the Arctic have facilitated range shifts by bringing seeds across dispersal barriers and increasing propagule pressure (Rew et al. 2020). For example, the increase in routes and maritime traffic for polar trade and tourism is expected to favour the arrival of non‐native species in remote polar areas (Huiskes et al. 2014; Seebens et al. 2018). In a comprehensive study of non‐native vascular plants in the Arctic, Wasowicz et al. (2020) identified seed contamination of luggage and equipment (tourism) and transport by vehicles (e.g., cars) as the major vectors of non‐native species introduction. In the high‐Arctic archipelago of Svalbard, heavy propagule pressure was associated with transport of seeds on tourist footwear, with nearly half of all visitors carrying at least one species and an estimated yearly load of ~270,000 seeds (Ware et al. 2012). Anthropogenic infrastructures at the local scale, such as trails and roads, may also increase the species geographic range and the propagule pressure of both native and non‐native plant species by reducing competition and/or facilitating the dispersal of propagules that cling to animals and hikers along the trail (Huiskes et al. 2014; Ware et al. 2012). Overall, the presence of human activity is expected to increase the probability of non‐native plant establishment and the alteration (including expansion) of native species distribution, thus defining high‐risk natural environments (Bartlett et al. 2021). In this sense, we define human disturbance as any human activity that changes habitat conditions (e.g., soil compaction, presence of competing vegetation) or increases dispersal ability or frequency (e.g., by bringing in and assisting the spread of seeds). High‐risk sites include natural habitats linked with ecotourism, such as landing sites accessible by boat, tourism infrastructures (e.g., basecamps) and hiking trails with high visitor numbers (Anderson et al. 2015). Although the redistribution of species is a mechanism to cope with the alteration of the living conditions due to climate change, it can also lead to very strong changes in local species compositions, in particular if highly competitive species establish and outcompete local species that are less able to shift their ranges due to migration barriers or lack of low competition habitats (e.g., at mountain tops or islands; Carroll et al. 2023). Thus, an important goal for research in the High Arctic is to understand and monitor the effect of human disturbance on vegetation change. To this end, the Svalbard archipelago can offer informative case studies due to its increasing vulnerability to extreme climate warming and anthropogenic disturbances. Namely, Svalbard has experienced one of the highest increases in temperature within the high‐Arctic environment, leading to fast ice melting and a lengthening of the growing season, and thus to a widening of the potential niche for many plant species (Hanssen‐Bauer et al. 2019; Lind, Ingvaldsen, and Furevik 2018; Nordli et al. 2014). Furthermore, Svalbard has recently become one of the most accessible regions in the polar area due to higher trading routes and tourism within the island, which increased both the risk of human‐driven IAS and range expansion of native species (Stocker, Renner, and Knol‐Kauffman 2020) and the need to efficiently monitor the vegetation change within the island (Bartlett et al. 2021).

To better understand how human activities might influence plant redistribution in a rapidly warming region, we conducted a spatial analysis on the arrival and spread of a native plant species, the Svalbard poppy (*Papaver dahlianum*), in a high‐risk natural environment of the Svalbard archipelago (Kapp Linné), where the species was absent prior to 1928. Specifically, we (i) conducted ground and drone surveys to monitor the abundance and terrain preference of *P. dahlianum* along the major hiking trail of the area, (ii) used statistical and modelling analyses to assess whether human disturbance (settlement and trail) favoured the introduction and spread of *P. dahlianum* and (iii) evaluated our results against historical occurrence records of the species. We hypothesised that the presence of human settlements and hiking trails facilitated the range expansion of *P. dahlianum* at Kapp Linné.

## 2 Methods

### 2.1 Study Site and Target Species

**FIGURE 1:**
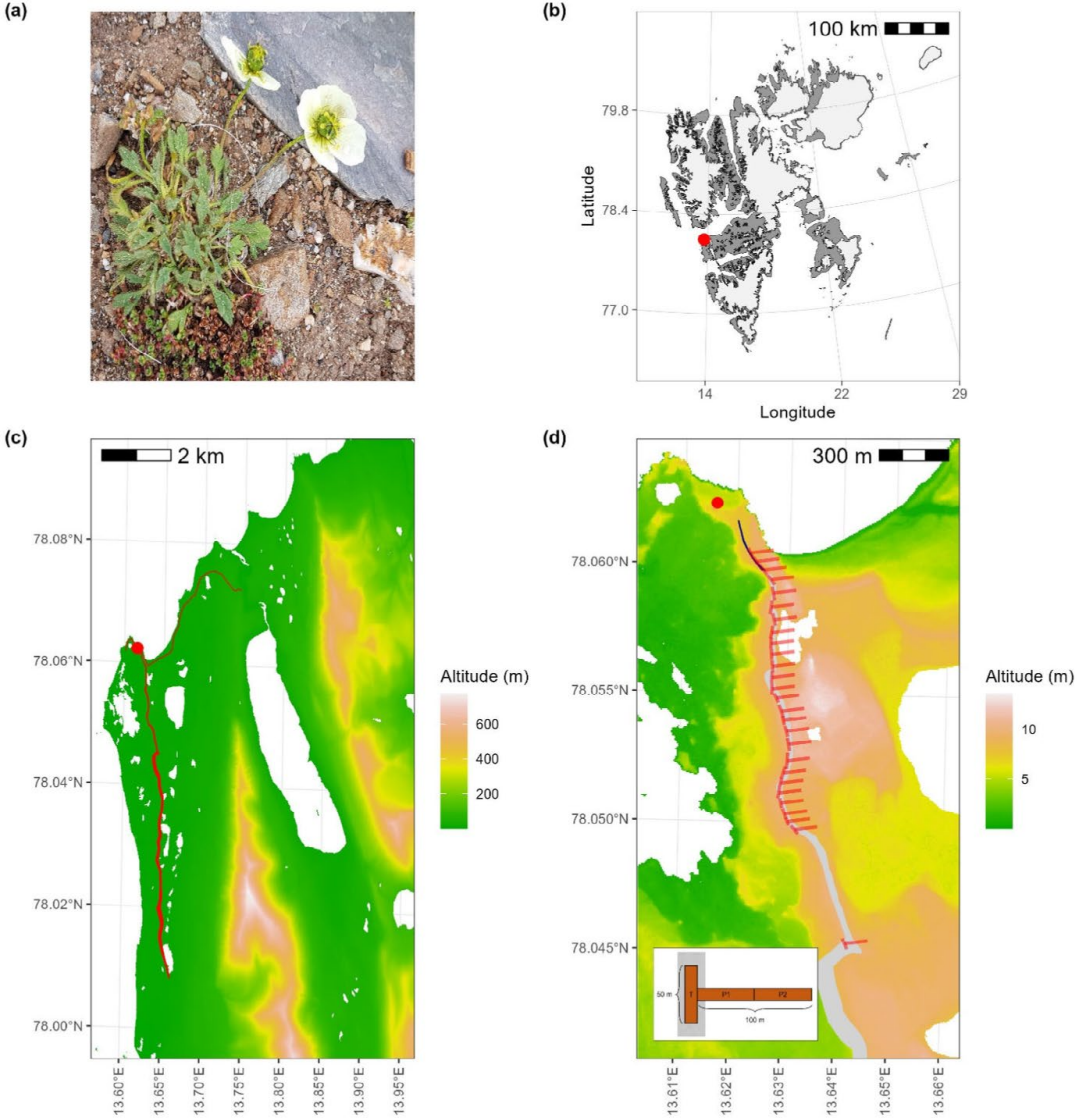
Study site and target species. (a) Photo of *Papaver dahlianum* specimen (2023 survey). (b) Location of the study area (red dot) in Svalbard. (c) Research area of historical landscape surveys (1994, 2014 and 2018) at Kapp Linné, with major hiking trails highlighted in red. (d) Surveyed area of July 2023 with the location of the 26 T‐shaped transects (each consisting of a 50‐m trail plot plus two perpendicular off‐trail plots; see insert) marked in red at an equal distance (~50 m) along the tractor track (dark blue) and the southward hiking trail (light grey). Red dots in (c) and (d) indicate the centre of the Isfjord Radio station (78°3′44.45″ N, 13°36′35.75″ E). The underlying maps for (b) and (c, d) were plotted using the R package ‘PlotSvalbard’ (Vihtakari 2020) and the DEM provided by the Norwegian Polar Institute (2014b), respectively.

We investigated the colonisation and spread of a native plant species in the area of Kapp Linné (78°04′ N, 13°38′ E) located in the northwestern‐most part of Spitsbergen (Figure 1b), the biggest island of the archipelago of Svalbard (Josefsson and Mårtensson 1995). This area is largely occupied by an intact natural habitat of numerous small dams, bog areas, rock outcrops, sets of raised beach ridges between 4 and 52 m a.s.l., the present beach deposits and slope colluvium (Hjelle and Ohta 1974). The area surrounding the investigated trail is a wide strand flat characterised by a set of raised beach ridges up to ~50 m a.s.l., rich in outcrops of metamorphic rocks, small lakes and ponds. Overall, the investigated trail follows the flat, continuous 8–12 m a.s.l. beach ridges (formed between 4500 and 6000 years BP). The soils of the ridges are dominated by a fairly uniform and well‐washed beach gravel material in the upper 1.5–3 m, where the well‐drained gravel shows an incipient pedogenetic development with a low amount of finer fractions and soil organic matter.

The main human activities are restricted to the Isfjord Radio station and the Kapp Linné lighthouse (from September 1933), a hotel established in 2000 in the still operating radio station buildings, and two main tracks starting from the station area (red dot in Figure 1c,d). These tracks are used by vehicular traffic for maintenance close to Isfjord Radio station (tractor tracks) or pedestrian traffic for tourist walks extending for kilometres to the east along the coast or southward to a trappers cabin and a recently established walrus colony (hiking trails) (red line in Figure 1c). Furthermore, the main part of the Kapp Linné and Fyrsjöen lake area is a bird sanctuary with entrance restrictions between April 15 and August 15. A thorough description of the geological and geomorphological settings at the site is given by Åkerman (1980).

The target species of this study is the symbolic flower of Svalbard, *P. dahlianum* Nordh., also called Svalbard poppy (Figure 1a). *P. dahlianum* is considered one of the hardiest forbs in high‐Arctic ecosystems and can be found on all major islands of Svalbard and in northeastern parts of Greenland and northern Norway. By persisting under marginal conditions (i.e., very poor soil conditions), this species experiences poor or no competition from other plants. *P. dahlianum* can grow in poorly vegetated rocky sites (e.g., moraines, screes and fellfields) and other disturbed areas such as roadsides (Elvebakk and Prestrud 1996). Cooper et al. (2004) showed how anthropogenic or natural disturbances (e.g., overgrazing, freeze–thaw action) enhance opportunities for the seedling establishment of *P. dahlianum*. Furthermore, this species is endowed with a great tolerance for freezing events (Körner and Alsos 2008) and can readily respond to climate warming by adjusting its flower production (Wookey et al. 1993). As a seed‐risking strategist (i.e., late flowering plant) with high freezing tolerance, *P. dahlianum* is fairly resistant to abrupt shifts to sub‐zero temperatures and to potential mismatches between flowering and pollination rates (being additionally self‐pollinating), which are two major threats to Arctic plant persistence under climate change (Kattsov et al. 2005). Consistently with its ecological strategy and plastic responses, this species appears to have expanded its range (from coastal areas to inner land) and increased its abundance over the last 50 years in the Isfjord area (Figure S1.1).

### 2.2 Historical and Contemporary Field Surveys

**FIGURE 2:**
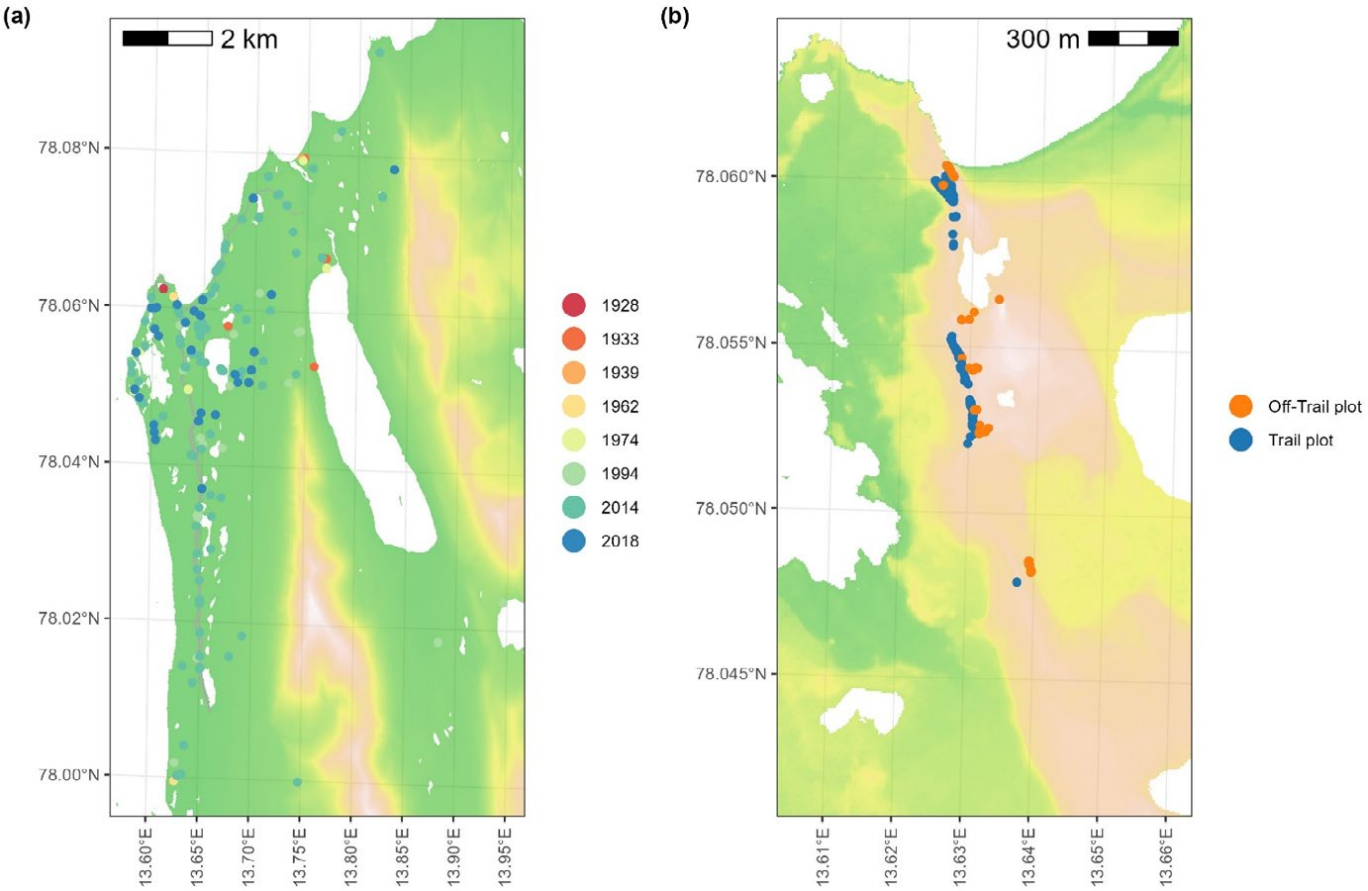
Observations of *Papaver dahlianum* according to (a) historical records at Kapp Linné (1928–2018) and (b) the 2023 survey along the southward hiking trail. Dots indicate species occurrence and are coloured by (a) year of first sighting or (b) position on the trail plots or on the perpendicular off‐trail plots. For the legend of underlying elevation maps, see Figure 1c,d, respectively.

Historical data on the distribution of the species as presence‐only coordinates were provided by long‐term monitoring of Kapp Linné starting from 1928, 1933 and 1939 (Hagen 1952), 1962 (P. Wold, unpublished 8 mm colour film), 1972 and 1974 (Åkerman 1983), 1994 (Josefsson and Mårtensson 1995) until 2014 and 2018 (J. Åkerman, unpublished) (Figure 2a). The earliest observations of the species presence and locations (1928, 1933, 1939, 1962, 1972 and 1974) were recorded in unsystematic field surveys (simple walks) of the area surrounding Isfjord Radio station. The first systematic survey of the research area (limits: 78°04′ N, 13°38′ E; 78°01′ N, 13°38′ E; 78°06′ N, 13°52′ E; 78°01′ N, 14°03′ E) was started in 1994 by T. Josefsson and I. Mårtensson for the production of a vegetation map of Kapp Linné (Josefsson and Mårtensson 1995), and continued by J. Åkerman in 2014 and 2018. In systematic surveys, the study area was subdivided in 1 × 1 km grid cells and the presence of *P. dahlianum* was recorded (with related GPS coordinates of the grid centre) in case one or more individuals were present within the grid cell.

We conducted ground surveys of *P. dahlianum* along the southward hiking trail starting from the Isfjord Radio station (Figures 1d and 2b). We modified the road survey protocol from the Mountain Invasion Research Network (MIREN) (Haider et al. 2022) by sampling the whole hiking trail at the same elevation. The first recorded individual was located at the trailhead in the proximity of the Isfjord Radio station, where the initial tractor track has higher disturbance due to the passage of vehicles. We placed T‐shaped transects on the trail at 50 m intervals starting at the trailhead, where each T‐shaped transect consists of three plots of 2 m width and 50 m length (insert in Figure 1d). The first plot (trail plot) was placed directly on the trail (and not on the trailside as in the original MIREN protocol), thus sampling the entire surface of the trail. The remaining two plots were placed perpendicularly to the trail one behind the other on the east side of the trail starting from the middle of the trail plot, thus forming a 100 m perpendicular plot (off‐trail plot). Off‐trail plots were only checked in an eastward direction due to the presence of a protected bird reserve westward of the trail. We kept sampling the trail until five consecutive trail plots were found devoid of *P. dahlianum*, for a total of 26 T‐shape transects (Figure 1d). Along the surveyed area, we recorded the position of *P. dahlianum* individuals with RTK‐corrected GPS coordinates (approximately 2 cm precision) and relative abundance (number of individuals within a 50‐cm radius from the GPS measurement). The trail and transects were surveyed in early July 2023 after the start of the flowering season.

### 2.3 Analysis of Human Disturbances on Observed Species Distribution

From written historical records (Section 2.2), the first sighting of *P. dahlianum* during 1928 was estimated to be located near the small lake Fyrdammen within the periphery of Isfjord Radio station (Hagen 1952). To test whether the observed spread of the species in later decades is consistent with this occurrence being the location of first colonisation, we calculated the species density as the probability distribution of either number of occurrences (historical surveys) or individual abundance (2023 survey) at increasing distances from the station (78°03′08″ N, 13°36′04″ E). Additionally, we estimated the spatial density of species presence in the research area starting from the point‐data of the last historical record (2018) using a kernel density estimation with Gaussian kernels (KDE) as implemented by the *gaussian_kde* function of the ‘scipy’ Python library. These approaches assume that the species density would be at its highest close to points of initial introduction. Our analysis tests whether the contemporary distribution of *P. dahlianum* at Kapp Linné can be the result of a single introduction at the start of an invasion process.

To understand the effect of trails on the density of *P. dahlianum*, we analysed the median count of species occurrence for the systematic historical records (1994, 2014 and 2018) on the hiking trails and sides (0–100 m from the trail centre), at 1 km distance from the trails (0.1–1 km) and farther away (> 1 km). For the contemporary survey, we compared the abundance of plants on the trail plot against the plant abundance on the perpendicular off‐trail plot using a paired Wilcoxon test (*n* = 26 T‐shaped transects).

We investigated the relationship between the density of *P. dahlianum* across the research area (Figure 2a) and the distance from the station (metres from 78°3′44.45″ N, 13°36′35.75″ E), the distance to the closest trail (in meters) and elevation (metres a.s.l.) by using a generalised linear model (GLM) with a Gaussian error distribution, using normalised variables. The species density was calculated as the normalised KDE from historical records of occurrence, whereas the elevation across the research area was extracted from the DEM provided by the NPI (Norwegian Polar Institute 2014b). The sample size of the GLM corresponds to the number of cells in the DEM of 5 m resolution (*n* = 3,024,470). We defined a full model by including all topographic and anthropogenic factors as explaining variables (predictors) of species density, including an interaction factor between distance from the station and distance from the closest trail. The interaction factor defines how proximity to the station influences the positive relationship between proximity to the trail and the occurrence of *P. dahlianum*. In other words, a high interaction factor verifies our hypothesis that the spread of the species in the study area was facilitated by the trail, where the historical spread started from the station. Additionally, we built sub‐models by progressively excluding specific variables or using each variable separately as a single explaining factor. In order to select the most informative GLM, we calculated the adjusted McFadden *R* ^2^, which represents the proportion of variance explained by all predictors included in each model, after adjusting for the number of predictors (Smith and McKenna 2013). Finally, we calculated the partial *R* ^2^ using the *rsq.partial* function of the ‘rsq’ R package (Zhang, Zhang, and Generalized R‐squared 2022) to determine the variation in *P. dahlianum* density explained by each of the predictors of the best GLM model.

### 2.4 Modelling of Species Spread (1928–2018)

We modelled the spread of the locally invasive species *P. dahlianum* in the research area of Kapp Linné (Figure 2a) starting from the year of the first observation until the last systematic landscape survey (1928–2018). We used a spatially explicit model on a grid with square cells of 5 m side length where the state of each cell of the simulation domain can be either occupied (1) or unoccupied (0), and updated each year based on an establishment kernel and topographic suitability. The 2D grid domain was derived from the Isfjord DEM of the Norwegian Polar Institute (2014b) and clipped to the limits of the research area (see Section 2.2). Topographic suitability (0/1) was based on the presence of water bodies, where cells with sea or lakes are unsuitable for establishment, and on an altitudinal upper threshold (~137 m as the highest observation in the dataset) above which *P. dahlianum* cannot establish. The position of water bodies was provided by the Norwegian Polar Institute (2014a) and the altitudinal suitability derived from a binomial GLM with the historical occurrence of *P. dahlianum* being a function of elevation. Given a suitable topography, establishment could potentially occur in unoccupied sink cells—0(*x*,*y*), where *x* and *y* are the longitudinal and latitudinal position of the cell, respectively—with a probability dependent on the distance from an occupied source cell, 1(*x*′,*y*′). This probability was defined by an establishment kernel, that is, the spatial distribution of established individuals relative to the source (also known as the effective dispersal kernel; Bullock et al. 2017). Whereas we used KDE as a non‐parametric method to estimate the probability density function (PDF) of the species occurrence based on a mathematical weighting function (Gaussian kernel), the establishment kernel describes the distribution of plant dispersal and establishment based on PDFs, where the PDF parameters define the shape and extent of the dispersal events. We used the abundance data collected from the contemporary survey along the southward hiking trail to fit two establishment kernels that differed in their assumption of an effect of trails as spreading corridors (including either a trail effect or no trail effect). We fitted the kernel of ‘local spread’ by assuming that the population build‐up in the immediate neighbourhood (density distribution of closest individuals either on the trail or outside) represents the local establishment per year without the trail effect. Alternatively, we included the trail effect by fitting a kernel of ‘trail spread’ under the assumption that individuals established outside of the trail have as a source of origin the closest individual on the trail. For the kernel fitting, we selected the probability distribution function (PDF) and related parameters based on the minimum sum square error (SSE) (and secondarily on the Bayesian information criterion [BIC]) by using the *Fitter* function of the ‘fitter’ Python library. We then applied the *mean* and *skew* functions of the ‘scipy.stats' Python module to calculate the mean distance of establishment and the skewness, respectively, for each fitted PDF. A high positive skewness was interpreted as a higher chance of rare events of long‐distance (LD) establishment, compared to the more common events at mean distances (Proença‐Ferreira et al. 2023).

Next, we used the best PDF and parameters under each assumption to generate establishment kernels in the 2D simulation grid by defining the distance between a source (*x*′,*y*′) and sink (*x*,*y*) cell as $z=\sqrt{{\left(x-x^{\prime }\right)}^{2}+{\left(y-y^{\prime }\right)}^{2}}$. The probability of establishment in a sink cell *P*_*e*_(*x*,*y*) was then defined by the convolution of the occupied cells and the establishment kernels, that is, the integral describing the spatial distribution of established individuals from the source as it is shifted over the locations of the established individuals. In order to decrease the computational load of the convolution, we applied the Fast Fourier Transform (FFT) method with the *fftconvolve* function of the ‘scipy’ Python library. To add a stochastic component to the species establishment, we generated random values scaled from zero to one for each cell and year of simulation, which represent a stochastic threshold for establishment at each cell, *T*_*e*_(*x*,*y*). In other words, the establishment kernel predicts a probability of establishment for each cell in each year, rather than a binary prediction of establishment or not. We randomly selected the threshold for each cell, *T*_*e*_(*x*,*y*), above which establishment would have occurred or not occurred:
(1)

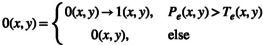




We initialised the simulations with a single occurrence according to the first historical record (78°03′44.45″ N, 13°36′35.75″ E in 1928). We then applied two separate spreading scenarios by using (1) only the kernel of ‘local spread’ for all source cells (‘no trail effect’) or (2) additionally the kernel of ‘trail spread’ if the source cell was on the trail (‘trail effect’). For each spreading scenario, we performed 10 replicate simulations and averaged the arrival time at each cell across all replicates.

Finally, we evaluated model simulations for each spreading scenario against historical occurrences recorded in the systematic landscape surveys (1994, 2014 and 2018). To do so, we calculated the model sensitivity as the probability of correctly identifying establishment sites according to observational data:

(2) $$\text{Sensitivity}=\frac{\mathrm{TP}}{\mathrm{TP}+\mathrm{FA}}$$

where TP is the number of correctly simulated presences (true presence) and FA is the number of incorrectly simulated absences (false absences), with respect to the observed values for each year (1994, 2014 and 2018). With this metric, we aimed to estimate the extent of the spread of the species outwards from the introduction point and not the correct locations of presences and absences within the area of occupancy. After excluding unsuitable areas for establishment by water presence and an elevation threshold, the only process underlying the spread and occupancy of *P. dahlianum* in our model is the species dispersal capacity as fitted by spatial data. Thus, we assume that our simulated range of occupancy (potential dispersal range) will have a potentially larger extent compared to the observed species distribution, where individuals are further subjected to microenvironmental conditions for survival and establishment.

### 2.5 UAV Data Collection and Analysis of Terrain Preference

In order to complement the ground survey with a fine‐scale overview of terrain types, we used an unmanned aerial vehicle (UAV) and generated a low altitude aerial image of the surveyed area along the southward hiking trail including all recorded presences of *P. dahlianum* (2023 survey) (see Appendix S1.A in Supporting Information).

Within a landscape, the occurrence of an individual at a site can be determined by both local dispersal processes and fine‐scale environmental conditions, such as terrain type. In this sense, the higher abundance of *P. dahlianum* individuals on hiking trails can indicate a preference for trampled terrains (e.g., low vegetation cover, and hence lower competition) and/or the soil substrate of the trail itself (e.g., gravel or with coarse material, due to erosion of this locally elevated area). To determine whether *P. dahlianum* individuals at Kapp Linné have a preference for a certain terrain type, we analysed the relationship between species presence and brightness of UAV images at a fine scale, where dark values are associated with relatively dense vegetation cover, whereas bright values indicate gravely terrains with sparse vegetation cover.

As index of brightness, we calculated the relative luminance [0–1] from the RGB channels (Caldwell et al. 2008) (see Appendix S1.B in Supporting Information). Next, we conducted a randomisation test, which accounted for spatial dependencies between the occurrences, by shifting the position of all observed poppies in north–south and east–west directions by a randomly chosen distance with a maximum of 10 m (higher values result in resampled points lying outside the UAV image) using a uniform distribution for both directions. We generated 1000 randomisations (with the first iteration being the observed positions). By shifting all individuals' positions by the same randomly drawn distances at each iteration, we aimed to maintain the spatial structure of the observed distribution, which is given by local dispersal processes, and thus analyse the contribution of terrain brightness (terrain type) in determining the occurrence of *P. dahlianum* individuals. We then calculated the median of luminance values, $\mathrm{med}\left(Y_{i}\right)$, for each random iteration i (with a total of n iterations = 1000) and obtained the proportion of randomisations in which the brightness of the terrain at the randomised positions is larger or equal to the brightness at the observed positions, $\mathrm{med}\left(Y_{0}\right)$. Given a large number of randomisations, this proportion is an estimator for the *p*‐value of the test whether the observed *P. dahlianum* individuals occur at locations with a higher brightness than expected by a random distribution.

(3) $$p-\text{value}≅\frac{\text{Count}\left[\mathrm{med}\left(Y_{i}\right)\geq \mathrm{med}\left(Y_{0}\right)\right]}{n}$$

As usual, a *p*‐value below the significance threshold (0.05) would indicate that there is a significant difference in the terrain brightness where *P. dahlianum* individuals occur compared to randomised occurrences; that is, it is unlikely that individuals grow on a specific terrain type only by chance.

## 3 Results

### 3.1 Drivers of Species Density: Distance From the Station, Trails and Topography

**FIGURE 3:**
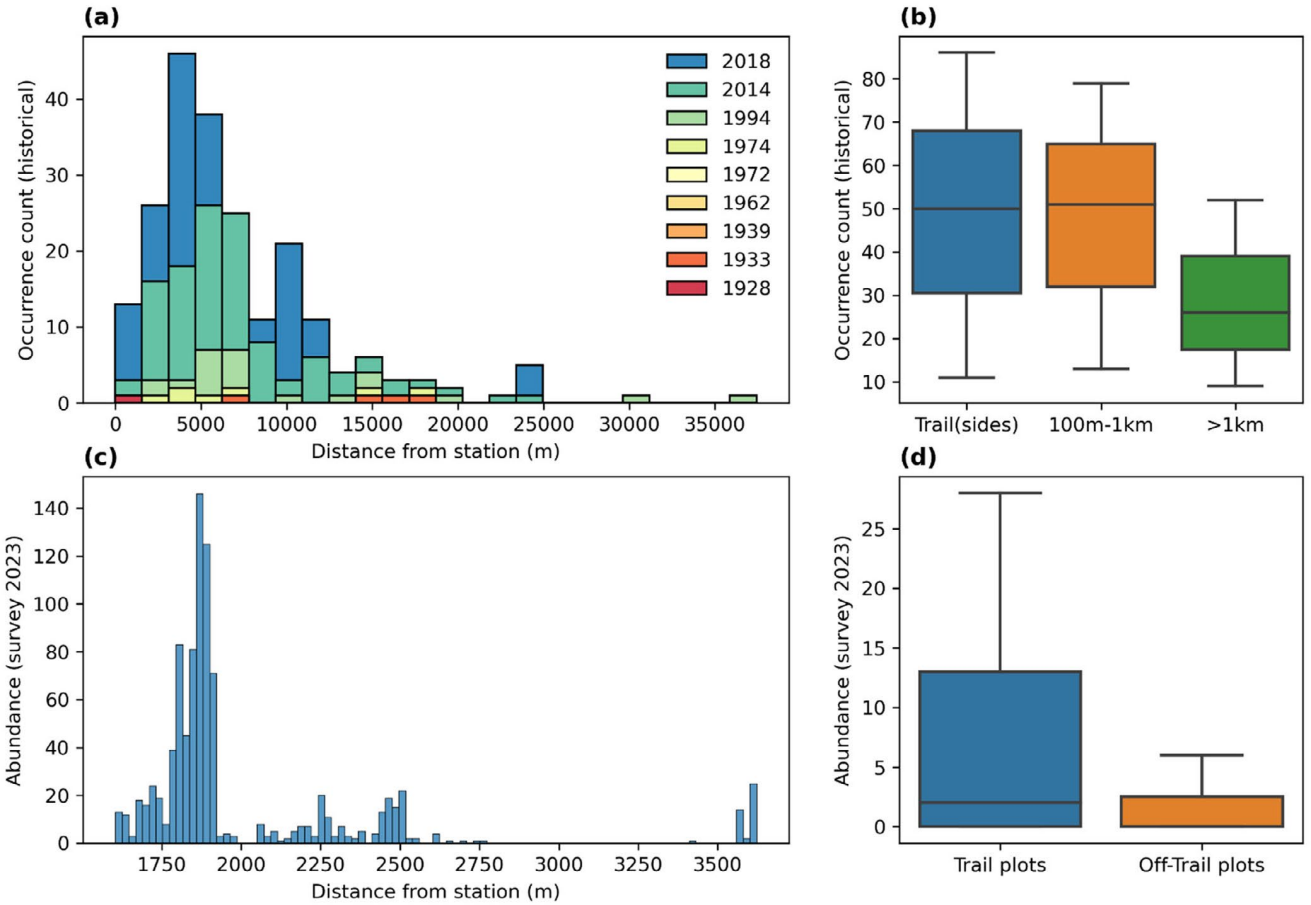
(a) Historical occurrences and (c) abundance of individuals (2023 survey) at increasing distances from the Isfjord Radio station (78°3′44.45″ N, 13°36′35.75″ E). Median and ranges of (b) occurrences on the trail (−sides) (0–100 m) or at increasing distances from the trail (0.1–1 km and > 1 km) for the complete historical records (1994, 2014, 2018) and (d) median abundance of individuals on the trail or adjacent transects per measurement point (*n* = 26) in the 2023 survey.

Our ground survey of *P. dahlianum* along the southward hiking trail of Isfjord Radio station showed a markedly higher proportion of individuals on the trail (86.9%; *n* = 624) than along the perpendicular plots off the trail surface (13.1%; *n* = 94) at relatively low level of disturbance, that is, human (and animal) trampling (Figure S1.2). Conversely, plants almost entirely grew on the trailside and along the off‐trail plots (99.1%) of the tractor track close to the station, with few individuals found on the trail (0.9%) possibly due to the high disturbance from vehicles (Figure S1.2). Consistently with the 2023 survey on the major hiking trail (Figure 3d), historical landscape surveys showed an overall higher species occurrence on all trails at Kapp Linné than that at increasing distances from the trail margins (Figure 3b). Specifically, species abundance on transects was on average two times lower on off‐trail plots compared to that on the major trail (Figure 3; Wilcoxon test, *p*‐value = 0.002), and trails had on average 1.6 times more *P. dahlianum* occurrence than that at regions more than 1 km away from any trail (Figure 3b). On the other hand, we observed equal numbers of occurrences on the trail and trailside (0.1–1 km from the trail) (Figure 3b).

The highest species abundance (and number of occurrences in historical surveys) was observed at a distance relatively close to the Isfjord Radio station, that is, ~2 (~5) km, followed by a sharp decline in species density at increasing distances from the station (Figure 3a,c). More precisely, the KDE reconstruction of the 2D spatial density indicated that the hot spot of the species occurrence was located at the bifurcation of the two tracks that start from the station, that is, between the trailheads of the eastward coastal trail and the southward hiking trail (cf. Figures 2a and S1.3d).

The GLM analysis confirmed that the variation in *P. dahlianum* density was mainly linked to anthropogenic factors (landing site location and trails) and, to a lesser degree, topography. Our best GLM model explained 75.5% of the observed variation in species occurrence by including all variables and the interaction factor between distance from the station and distance from the closest trail (‘full model’) (Table S1). Partitioning the variance of our best model, distance from the station captured the highest partial variance (56.1%), followed by the interaction factor (38.6%) and distance from the closest trail (27.5%) (Table 1). Altitude accounted only for 1.5% of the variance in our best GLM model (Table 1). Accordingly, the lowest explained variance (17.8%) was obtained from the sub‐model using altitude as the only predictor of species density (sub‐model 5; Table S1). In other words, the occurrence of *P. dahlianum* was positively influenced by the proximity to the trail and to the station, where the positive effect of the trail's proximity on the species occurrence was enhanced the closer the trail was to the station.

**TABLE 1:** Parameters of the best generalised linear model (GLM) model (family Poisson) for the density of *Papaver dahlianum* as normalised kernel density estimation (Gaussian kernel) from historical records (no. of observations = 3,024,470). Predictors include elevation (in metres), distance from the station and distance from the closest trail (Euclidean). The explained variance for each predictor is the percentage of the partial *R* ^2^. The full *R* ^2^ (McFadden) of our best GLM model is 0.7548. For the explained variance of the remaining models, see Table S1. As it is common practice *** stands for the significance level *p* < 0.001.

	Estimate	Std. Error	*z* value	Pr(>|*z*|)	Explained variance [%]
Intercept	0.66	0.0002	3122.3	***	
Distance from station	−1.06	0.0005	−1964.4	***	56.1
Distance from station to trail	1.49	0.0011	1379.0	***	38.6
Distance from trail	−0.92	0.0009	−1069.8	***	27.5
Elevation	−0.06	0.0003	−213.1	***	1.5

### 3.2 Best‐Fitted Kernel of Species Occurrence

The top‐5 establishment kernels fitted from the *P. dahlianum* abundance of the 2023 survey were selected based on the lowest SSE (and further confirmed by BIC) for both spreading assumptions, that is, when assuming either local spread (i.e., the closest individual is the source of spread; Figure S1.4a) or spread from the trail (i.e., the source is the closest individual on the trail; Figure S1.4b) (Table 2). We found four common PDFs defining the top‐5 establishment kernels for both spreading assumptions (log‐normal, Cauchy, exponential and exponential power), although the shape of the best‐fitted kernels differed between local and trail spread. The best kernels of local spread and the observed establishments from which we fitted showed a sharp decline starting close to the source (~1–2 m) (Table 2 and Figure S1.4a). On the other hand, kernels built by assuming spread from the trail showed a peak of establishing individuals relatively farther away from the source (~30 m) with outliers as far as ~100–200 m (Table 2 and Figure S1.4b). Considering the best‐fitted kernels (lowest SSE and lowest BIC) with defined means (log‐normal PDFs, whereas Cauchy does not define any mean), the average distance of species establishment was ~15 times higher when the trail acted as a spreading source (~29.77 m) than when the local build‐up of individuals was fitted (~1.83 m) (Table 2). However, the importance of LD events as represented by a high positive skewness (or by the shape parameter when defined by the PDF) was overall higher for the establishment kernels fitted under the assumption of local spread (Table 2). In other words, species spread occurring from trails is faster in common establishment events, whereas local spread, despite being slower on average, might promote species spread through rare LD establishment events.

**TABLE 2:** Top‐5 kernel fits from contemporary abundances of *Papaver dahlianum* (survey of summer 2023) along the main hiking trail starting from the Isfjord Radio station. Kernels were fitted either assuming local spread (source is the closest individual) or spread from the trail (source is the closest individual on the trail). Best parameters for two‐parameter PDFs: location and scale and three‐parameter PDFs: shape, location and scale. PDF = probability density function. SSE = sum of squared errors (the sum of squared differences between the predicted establishment distance and the observed establishment distance). BIC = Bayesian information criterion.

Kernel	PDF	SSE	BIC	Best parameters	Mean distance	Skewness
Local spread	Log‐normal	0.0047	−4779.9	0.8611, −0.0799, 1.3177	1.83	10.64
	Cauchy^a^	0.0252	−4081.7	0.9121, 0.3121	0.58	13.65
	Exponential	0.0325	−3974.0	0.0014, 2.2614	2.26	8.5
	Exponential power	0.1396	−3354.7	0.4154, 0.0014, 16.4779	8.91	6.07
	Chi‐square	0.1651	−3284.1	1.5352, 0.0014, 5.2370	8.04	4.98
Trail spread	Cauchy^a^	0.0024	−4551.3	22.2442, 10.0405	22.24	2.23
	Log‐normal	0.0026	−4518.6	0.5760, −9.9562, 33.6560	29.77	1.49
	Exponential power	0.0029	−4475.3	0.7218, 0.4961, 52.6296	29.60	2.37
	Rayleigh	0.0029	−4477.6	−9.9771, 33.7059	32.27	0.97
	Exponential	0.0031	−4456.7	0.4961, 29.3429	29.84	1.94

^a^ As the mean is not defined in the Cauchy distribution, we report the median (=location parameter).

### 3.3 Trail Effect on Modelled Species Spread

**FIGURE 4:**
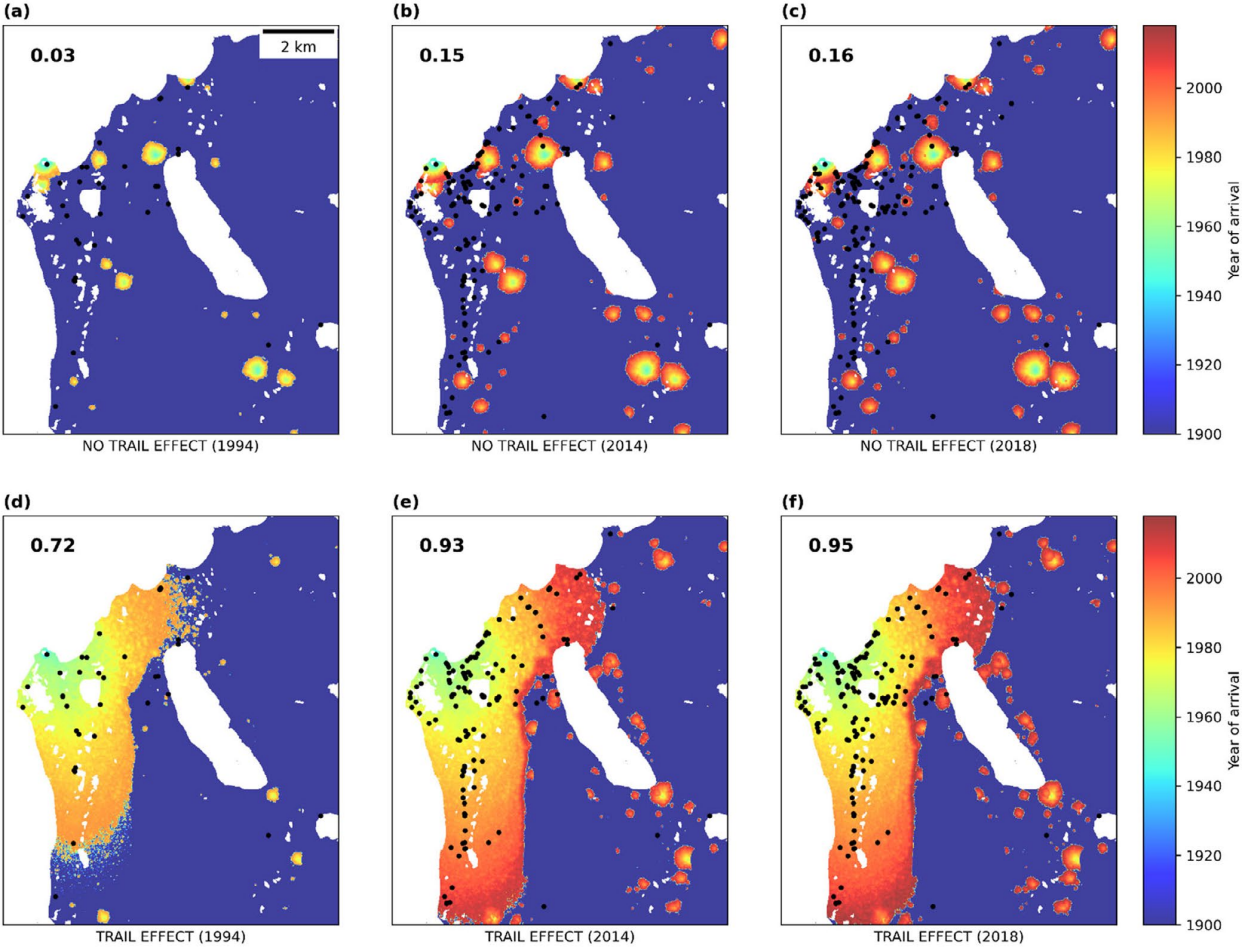
Simulated arrival of *Papaver dahlianum* (i.e., first year of establishment) at 3 years of systematic landscape survey—1994 (a, d), 2014 (b, e) and 2018 (c, f)—with related historical presences (black dots), and for two spreading scenarios: (a–c) assuming only local spread or (d–f) an additional trail effect (i.e., higher establishment probability if spreading from the trail). Model sensitivity for each simulated year (i.e., proportion of true presences) is reported in the upper left corner. Dark blue indicates areas without species establishment. Total temporal domain: 1928–2018. Spatial resolution: 5 m. Simulation replicates: 10 (replicate averages are reported in the panels).

The simulation outcomes of the potential establishment of *P. dahlianum* (1928–2018) strongly differed based on the spreading scenario, that is, by either applying only the best‐fitted kernel of local spread (‘no trail effect’; Figure 4a–c), or by additionally combining it with the best‐fitted kernel of spread from the trail (‘trail effect’; Figure 4d–f). Specifically, the modelled species establishment assuming exclusively close neighbours as a source of spread (‘no trail effect’) performed poorly when trying to explain the wide distribution of *P. dahlianum* in the area of Kapp Linné (Figure 4a–c). This was reflected by extremely low sensitivity values (i.e., very few correctly simulated presences) for the 3 years of landscape survey (sensitivity ≤ 0.2 for 1994, 2014 and 2018; Figure 4a–c). In agreement with the above‐described behaviour of “local spread” kernels (high probability of establishment close to the source with rare colonisation events in the tail extremes; Section 3.2), the majority of simulated true presences were located around the initialisation point (1928, Isfjord Radio station). Less frequent but LD establishment events still allowed some individuals to reach far‐off sites of observed presence. Nevertheless, rare LD events were not sufficient to promote the spread of the species across the cluster of observed presences stretching southward and eastward from the station (Figure 4a–c). Conversely, the model scenario where species establishment was promoted by a faster spread from the trail (‘trail effect’) managed to capture most of the observed area of occupancy, with markedly higher rates of correctly simulated presences across the last decade (sensitivity ≥ 0.9 for 2014 and 2018; Figure 4e,f). Similarly, to the scenario with ‘no trail effect’, the colonisation of isolated and distant sites was enabled by rare LD events according to the establishment probabilities of the ‘local spread’ kernel. Additionally, faster spread from the two main trails leading southward and eastward from the station likely leads to the large and continuous L‐shaped range of occupancy under the ‘trail effect’ scenario, which includes the bulk of observed presences (Figure 4d–f).

### 3.4 Fine‐Scale Terrain Preference

**FIGURE 5:**
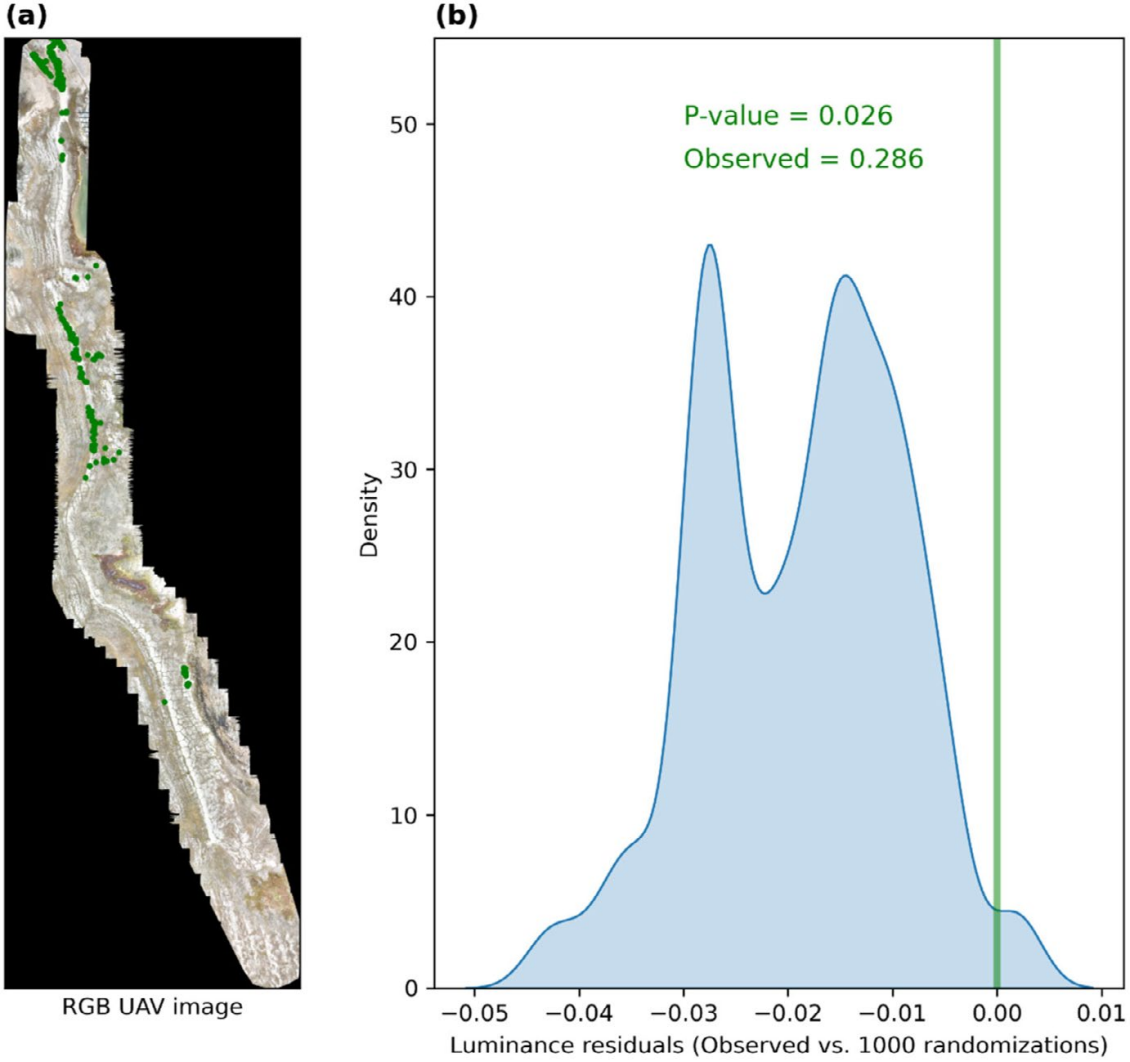
UAV survey along the major hiking trail. (a) RGB image with observed presence of *Papaver dahlianum* (green dots) and (b) distribution of residuals between observed (median reported in green) and randomised luminance values, 0–1 (1000 randomisations). (a) Spatial resolution: 10 cm. For details on randomisation technique, see Section 2.5.

The randomisation test maintaining the spatial structure and using terrain brightness at 10 cm resolution as a proxy for fine‐scale substrate type showed that the species has a statistically significant preference for areas with higher luminance (*p*‐value < 0.05) (Figure 5b). These areas correspond to terrain with low amounts of exposed soil or vegetation because the dominant gravel is brighter than either the exposed soil or most plant cover (Figure 5a).

## 4 Discussion

### 4.1 Landing Sites as Introduction Points

We showed that a spatial analysis of species presence from a single time point can roughly identify the neighbourhood of the introduction point (Figure 3a,c). Namely, the highest concentration of observed occurrences or highest abundance of individuals should be located close to the introduction point. This assumes that individuals at the spreading front (or range edge) are more scattered and less abundant than those at the source (or range centre), that is, abundance core. More precisely, as range expansion from a source is a temporal process that requires individual growth, dispersal and population build‐up further away, the introduction point is expected to be the area with the highest population build‐up and thus to coincide with the abundance core (Fristoe et al. 2023). Applying the concepts of abundance dynamics to the dynamics of species spread, this assumption can be understood according to the ‘abundant‐centre’ or ‘abundant‐core’ hypothesis, where the abundance of a species should decline with increasing distance from the estimated core of abundance, that is, in our case the point of introduction (Arim et al. 2006; Fristoe et al. 2023). Our analysis of the spatial distribution and abundance of *P. dahlianum* at Kapp Linné agrees with this hypothesis. We found that distance from the station (i.e., the potential introduction point) explained the highest partial variance (56.1%) of the observed species density in our best GLM model (Table 1). Similarly, linear regression analysis indicated a negative and significant (*p*‐value < 0.0001) relationship between species occurrence and distance from the station. Thus, we may reasonably assume that the landing site of Isfjord Radio station was the introduction point of *P. dahlianum* in the area of Kapp Linné.

This is also supported by the spatial distribution of *P. dahlianum* derived from GBIF observations (1900–now), where the highest records of species occurrence are reported close to boat landing sites and ports across Isfjord (Figure S1.1b). In recent decades (2000–now), the highest concentration of *P. dahlianum* was located around the centre and the neighbourhood of Longyearbyen, which is the most densely populated area in the region and equipped with a huge port connected to the mainland and to smaller boat landing sites in the interior of the fjord (Figure S1.1b). Closer to our study area, both GBIF data and personal observations identified other clusters of *P. dahlianum* individuals in the vicinity of a tourist and research spot, that is, the glacial lake Linnévatnet (78°03′ N, 13°50′ E) and its corresponding landing sites, Russekeila and Soloveckijbukta. In the broader context of Isfjorden, *P. dahlianum* appears to have increased its abundance by spreading from coastal areas close to ports and landing sites to the inner land, especially from the beginning of the 21st century. Although present in the area as a native plant before 1950, it is likely that the species spreads more easily around Longyearbyen due to increased human disturbances (high visitor numbers and shipping pressure) and later colonised more remote touristic spots in the fjord, such as Kapp Linné. Although climate warming is quite extreme in the High Arctic, the colonisation and spread of *P. dahlianum* was most likely not a result of warming, given its historical presence in other parts of the Svalbard archipelago even at higher elevations (Figure S1.1). It is possible that the species might have occupied the area of Kapp Linné sooner if dispersal barriers were broken earlier by human presence and activities.

Previous studies have shown how human disturbance, such as settlements and landing events, can act as introduction points for IAS in Svalbard and other polar regions. For instance, experts in invasion biology, taxonomy and Arctic environments identified vessel traffic as a potential dispersal vector for invasive marine organisms on Svalbard (Cottier‐Cook et al. 2024). Similarly, a systematic survey of high‐risk habitats on Svalbard revealed the presence of 36 non‐native plant species with the highest concentration in areas affected by human activity (landing sites with high visitor numbers) and with nutrient‐rich soils through historical animal husbandry or the guano of bird cliffs (Bartlett et al. 2021).

### 4.2 Trails as Spreading Corridors

Our kernel analysis revealed a marked role of hiking trails in promoting the spread of *P. dahlianum* at Kapp Linné. Specifically, species establishment occurred ~15 times faster when assuming that trails might act as a source of spread (henceforth, ‘trail effect’; ~30 m per year) than when assuming exclusively local build‐up through natural spread (~2 m per year). The average distance fitted under the assumption of local (natural) spread is consistent with a passive dispersal mechanism, such as the primary vector of *P. dahlianum*, that is, ballistic seed dispersal. On the other hand, the high positive skewness of the fitted establishment kernel is compatible with LD events through its secondary dispersal mechanisms, that is, via wind (SVALBARDFLORA. 2020). Furthermore, we noted that the spatial distribution of abundance and presence of the species in the larger research area matches the shape of the establishment kernel fitted by assuming a trail effect (cf. Figures 3a,c and S1.5b), indicating that trail presence is a key determinant of the species distribution at Kapp Linné. In agreement with the kernel analysis, our best GLM model identified distance from the trail as the third best explanatory variable (27.5%) of *P. dahlianum* density, after distance from the station (56.1%) and their interacting effect (38.6%). Finally, we observed a far higher performance of the modelled species spread when accounting for fast dispersal from the trail (sensitivity ≥ 0.9) compared to the under‐prediction of species presence when including only local (natural) dispersal (sensitivity ≤ 0.2) (Figure 4). Taken together, these analyses suggest that the fast spread of *P. dahlianum* at Kapp Linné was likely promoted by the presence of hiking trails in the area.

The extensive MIREN survey (Liedtke et al. 2020; Alvarez et al. 2022; Haider et al. 2022) has shown how trails and roads may act as spreading corridors for non‐native plants by reducing competition, changing abiotic conditions and/or increasing the propagule pressure in mountainous regions (Iseli et al. 2023; Wedegärtner et al. 2022). Similarly, Lembrechts et al. (2016) highlighted the importance of human disturbance, and specifically roads, as sources of plant invasions in cold environments, suggesting that roads may act not only as spreading corridors via propagule transport but also as competitor‐free habitats, which are markedly less resistant to establishment of new individuals. For example, trail construction and/or trampling along the trail can remove the biomass of less resistant or smaller established individuals, thus allowing newly arrived species to colonise a trail habitat with low competition (Wedegärtner et al. 2022). This would be especially true for species limited by competition (weak competitors) and disturbance‐associated species. In this sense, *P. dahlianum* adopts the ecological strategy of pioneer plants, where the species is well adapted to harsh polar environments and prefers poorly vegetated sites with little or no competition (Daniëls et al. 2016; SVALBARDFLORA. 2020). Thus, the eradication of less‐sturdy plants along the trail by trampling would likely favour the growth of *P. dahlianum*. Furthermore, trails can act as spreading corridors by promoting the transport of propagules (e.g., seeds) throughout the path. Dispersal vectors can be either humans or animals that transport propagules on clothes and footwear of hikers (Huiskes et al. 2014; Ware et al. 2012), or on feet and fur of animals (epizoochory) and inside faeces (endozoochory) (Anne Bråthen et al. 2007; Green et al. 2018; Lovas‐Kiss et al. 2023). Because the fruits of *P. dahlianum* are rather durable, propagule dispersal under the footwear of hikers or the hooves of reindeers seems possible. On the other hand, grazing can damage and reduce the output of seeds, making endozoochory a less viable dispersal strategy for *P. dahlianum* (cf. *Papaver radicatum* in Graae, Pagh, and Bruun 2004).

In summary, trailheads located close to landing sites may act as introduction points for new individuals, which can then spread rapidly along hiking trails (Anderson et al. 2015; Pickering, Bear, and Hill 2007). Given the configuration of the tourism infrastructure at Kapp Linné (boat landing site close to a basecamp and two major trails), we conclude that human disturbance significantly contributed to the colonisation and spread of *P. dahlianum* in the area.

### 4.3 Fine‐Scale Distribution Pattern

The spread and the occurrences of species often follow different patterns (and ecological factors) at different scales. We first analysed the (relatively) large‐scale distribution pattern of *P. dahlianum* at Kapp Linné in relation to the position of a likely introduction point (landing site) and spreading corridor (hiking trails) in the area. In our UAV survey, we further investigated whether fine‐scale processes and spatial configuration might interact with the larger pattern of the species distribution. We thus generated an orthophoto at fine resolution (10 cm), where the brightness index reflects the proportion of terrain not covered by soil or plants (which are typically smaller, that is, in the order of 1–2 cm diameter) because the dominant gravel is brighter than both the exposed soil and most vegetation. This analysis showed that the preferred habitat type of *P. dahlianum* at Kapp Linné consists of terrains with rocky soils and/or with low vegetation cover. This terrain type is very prominent along the hiking trail, which was formed after the erosion of the top strata of an old beach ridge. The coarse material on the surface creates a rocky environment with limited soil, making the terrain less suitable for the establishment for more resource‐demanding plants and hence more suitable for the pioneer *P. dahlianum*. Indeed, the species has already been observed to prefer habitats with relatively well‐drained terrains, mixed or coarse substrates and low competition (SVALBARDFLORA. 2020). As *P. dahlianum* is quite resistant to harsh weather (e.g., strong wind and snow cover during winter), it can also grow on exposed or unprotected areas and disturbed sites, including fellfields, moraines, exposed plateaud, unstable slopes, screes and river bars. Thus, the species might prefer trails not only because they provide a relatively competition‐free habitat due to trampling but also because the substrate naturally favours low vegetation cover and even more so with increasing human disturbance. That is, trampling in such a sensitive arctic environment is highly damaging to non‐adapted, competing vegetation due to increased erosion. Wind and freezing/thawing cycles can increase the erosion in dry and wet areas, respectively, especially where less‐sturdy plants have been removed by trampling and the soil is more exposed. In turn, damaged plants may take a long time to regrow or not grow at all due to the current low productivity of the landscape, leaving the terrain still more vulnerable to erosion and hence suitable only for well‐adapted species, such as *P. dahlianum*. Overall, we cannot disentangle the trail effect from a natural preference for a certain terrain because the use of an area as a trail stems from similar properties of naturally suitable environments. For instance, the beach ridge is more frequently used as a hiking trail, while being also a naturally exposed area where the coarse material is deposited by waves, leading to a higher habitat suitability for the species. More generally, elevated and rocky areas are easier to walk upon, and provide a better overview (e.g., to spot polar bears) and a good reference point in an otherwise rather featureless landscape.

Finally, we conclude that the spreading rate of *P. dahlianum* at Kapp Linné is a combination of the availability of micro‐scale suitable habitats (given by the natural coarse substrate with low competition, further enhanced by trampling) and active transport from human activity.

### 4.4 Vulnerability and Management of IAS at Svalbard

The sparsity of human presence and extreme climatic conditions of polar environments tend to concentrate on the establishment of new species in human‐related ‘hot spots’ with higher propagule pressure and lower environmental harshness. This is especially the case for remote high‐Arctic islands such as the Svalbard archipelago, which have additional geographic barriers to the natural dispersal of plant species, and thus depend more on anthropogenic activities and disturbances for the introduction and spread of both native and alien species (Lembrechts et al. 2016). Factors facilitating the success of species establishment at Svalbard include a decrease in environmental harshness given by climate warming and an overall increase in economic activities, such as higher traffic of fishing and touristic vessels (cruise liners) (up 25% from 2013 to 2019), bigger vessels with more passengers (up 73% from 2008 to 2018), more abundant landing sites and a longer ‘touristic season’ (Stocker, Renner, and Knol‐Kauffman 2020).

Although we analysed the spread of a native species initially not present at the site (local spreader) and showed how human activities facilitated its spread, the same mechanisms are likely valid for alien species. Indeed, recent monitoring studies on alien vascular plants (Bartlett et al. 2021) and marine species (Cottier‐Cook et al. 2024) have shown that increasing human activities on Svalbard are linked to an increasing threat of biological invasions from IAS (introduced alien species). Given the increasing vulnerability to IAS and climate warming, a number of initiatives were developed in the last decades for the protection of polar environments. For instance, the Circumpolar Biodiversity Monitoring Programme (CBMP) (Christensen et al. 2013) and the Arctic Invasive Alien Species (ARIAS) initiative (CAFF‐PAME 2017) urged the development of action plans for the prevention and management of IAS in the Arctic. The effectiveness of such plans often relies on efficient methods for the detection of IAS presence and the evaluation of IAS risk. One promising methodology—the Early Detection and Rapid Response (EDRR) management (Waugh 2009)—aims at implementing a systematic monitoring plan to detect new IAS events, backtrack introduction points and spreading patterns, and identify potential invasive species before establishment and “hot spots” of IAS activity. Answering the calls from CBMP and ARIAS, Bartlett et al. (2021) identified IAS “hot spots” on Svalbard as high‐risk environments linked to human activities and presented an EDRR methodology based on UAVs and ground surveys to efficiently monitor the presence and dynamics of IAS in polar environments.

In support of the EDRR management, the low‐cost methods used in this study can be applied with a similar aim. Because human settlement or trails/tracks have a high chance of acting as spreading corridors for alien plants, a spatial analysis can be used to plan an efficient monitoring campaign. In this sense, the standardised product between distance from potential introduction points (e.g., ports) and distance from potential spreading routes (e.g., roads) can be used to derive the probability of encountering an alien species (i.e., risk map) in order to scale down the surveyed area. Ground surveys can start from ‘hot spot’ areas (i.e., highest values in the risk map) and continue along potential corridors of spread. To expand the monitoring beyond the sampled area, spatially explicit models can then be applied to predict (or backtrack) the potential spread of a species after parametrisation of the dispersal potential from the ground survey. In our case, a model based on establishment kernels fitted on one‐time‐point spatial data can be used to provide a potential risk map based on the dispersal capacity of the species. This model may predict the extent of the area with the highest likelihood of the species dispersing and establishing, although the rare events of LD dispersal will remain difficult to predict with precision (see black dots in blue areas; Figure 4d–f). Finally, using UAVs (e.g., calculating brightness index) can help to detect suitable areas at a finer scale based on landscape heterogeneity or other terrain features. Depending on the heterogeneity of the landscape, this information might even be applied to satellite images with, for example, brightness filtering to further refine the risk map of the target species across a larger area.

## 5 Conclusions

Our findings show that human disturbances, such as boat landing sites and hiking trails, may significantly facilitate the spread of a native plant species in polar environments. The dispersal facilitation was likely a result of active propagule transport by humans (e.g., seeds carried under the footwear of hikers) and the availability of microenvironmental conditions for establishment given by the presence of a favourable natural substrate and human trampling, where both reduced competition from less adapted and more resource‐demanding species. Nevertheless, the same mechanisms favouring the range expansion of a well‐adapted native plant may also allow the invasion of alien species under release of competition and climate warming. In this context, high‐Arctic natural environments linked with human activities should be monitored to identify potential IAS and community reassembly that might affect important ecosystem function, such as carbon sequestration. Our method can be used to identify entry points in order to apply sanitisation measures and minimise the monitoring effort in case of a planned eradication of invasive species.

## Author Contributions

**Deborah Zani:** conceptualization (equal), funding acquisition (lead), methodology (lead), writing – original draft (lead), writing – review and editing (equal). **Heike Lischke:** conceptualization (equal), project administration (equal), supervision (equal), writing – review and editing (equal). **Jonas Åkerman:** resources (equal), writing – review and editing (equal). **Veiko Lehsten:** conceptualization (equal), project administration (equal), resources (equal), supervision (equal), writing – review and editing (equal).

## Conflicts of Interest

The authors declare no conflicts of interest.

## Supporting information


Data S1.

## Data Availability

The data and code that support the findings of this study are openly available in the Zenodo repository at https://doi.org/10.5281/zenodo.12663291.
