# Supplementary material for: Hiking Trails Facilitate the Spread of a Native High‐Arctic Species
Source: Ecol Evol. 2025 Jan 9;15(1):e70809. doi: 10.1002/ece3.70809 (PMC11717554; doi:10.1002/ece3.70809)
Supplement: Supplementary file 1 — Data S1. [file ECE3-15-e70809-s001.docx]

**Appendix S1**

**Effect of human disturbance on the spread of a high-Arctic species**

**Appendix S1.A: UAV survey**

The UAV, a DJI Phantom 4 RTK, was equipped with an RGB camera and an RTK (real-time kinematic) module for high-resolution positioning. The flight plan was developed on the UgCS PRO software for UAV mission planning (v.4.16) with terrain correction (built-in elevation layer "North of 60deg N latitude") at 50 m height, a flight speed of 1.5 m s^-1^, and a forward and side overlap of 70% to decrease blurring effects. In order to survey the whole area, two flight sessions were conducted on the 11 and 15 July 2023 with similar light conditions. A total of 617 images were collected with RTK correction (CPOS, Mountpoint: SVALBARD), resulting in a horizontal accuracy of 5 cm. The high-resolution RGB images were then merged in a single orthophoto by using the open source software WebODM (Vacca, 2020). The ortophoto was generated in the WGS 84 UTM 33N projection (EPSG:32633) within the following boundaries: 78°3′36.94″N, 13°37′18.11″E; 78°2′33.08″N, 13°37′25.37″E; 78°2′33.54″N, 13°39′1.08″E; 78°3′37.40″N, 13°38′53.96″E. For the analysis of relative luminance, we downscaled the image from 5 cm to 10 cm resolution since this is roughly the size of a plant.

**Appendix S1.B: Relative luminance**

As index of brightness, we calculated the relative luminance [0-1] from the RGB channels (Caldwell et al., 2008). First, we divided the 8-bit numbers of each channel by 255 to normalize the initial RGB values of range [0-255] to the range [0-1]. As the RGB values are gamma-encoded (i.e. light is encoded with a power curve in the color space of the orthophoto), we derived the linear (or gamma-expanded) values of each channel as follows:

$C_{lin}=\left\{ \begin{aligned} \frac{C_{rgb}}{12.92}, &C_{rgb}\leq0.04045 \\ \left( \frac{C_{rgb}+0.055}{1.055} \right)^{2.4}, &C_{rgb}>0.04045 \end{aligned} \right.$ (S1)

where $C_{rgb}$ is the channel in the range 0 to 1 (either R, G, or B), and $C_{lin}$ is the gamma-expanded channel value. Relative luminance $Y$ [0-1] was then obtained by multiplying each channel by its coefficient of spectral weighting and summing up the three weighted channels:

$Y=0.2126\times R_{lin}+0.7152\times G_{lin}+0.0722\times B_{lin}$ (S2)

**Table S1**: Predictors and explained variance of generalized linear model (GLM) models for the density of *Papaver dahlianum*. Species density is a normalized Kernel Density Estimation (Gaussian kernel) from historical records (no. observations=3,024,470, i.e. number of cells in the DEM of 5 m resolution provided the Norwegian Polar Institution, 2014b). Predictors include elevation (in meters), distance from the station and distance from the closest trail (Euclidean). The explained variance is calculated as the percentage of McFadden R^2^ (adjusted by the number of predictors).

| **Model** | **Predictors** | **Explained variance [%]** | **SSE** |
| --- | --- | --- | --- |
| *Full model* | $\sim$ Distance from station : trail + Distance from station + Distance from trail + Elevation | 75.5 | 23680.79 |
| *Sub-model 1* | $\sim$ Distance from station : trail + Distance from station + Distance from trail | 75.1 | 24036.40 |
| *Sub-model 2* | $\sim$ Distance from station + Distance from trail | 57.3 | 41245.18 |
| *Sub-model 3* | $\sim$ Distance from station | 56.9 | 41607.23 |
| *Sub-model 4* | $\sim$ Distance from trail | 38.8 | 59099.73 |
| *Sub-model 5* | $\sim$ Elevation | 17.8 | 79441.40 |


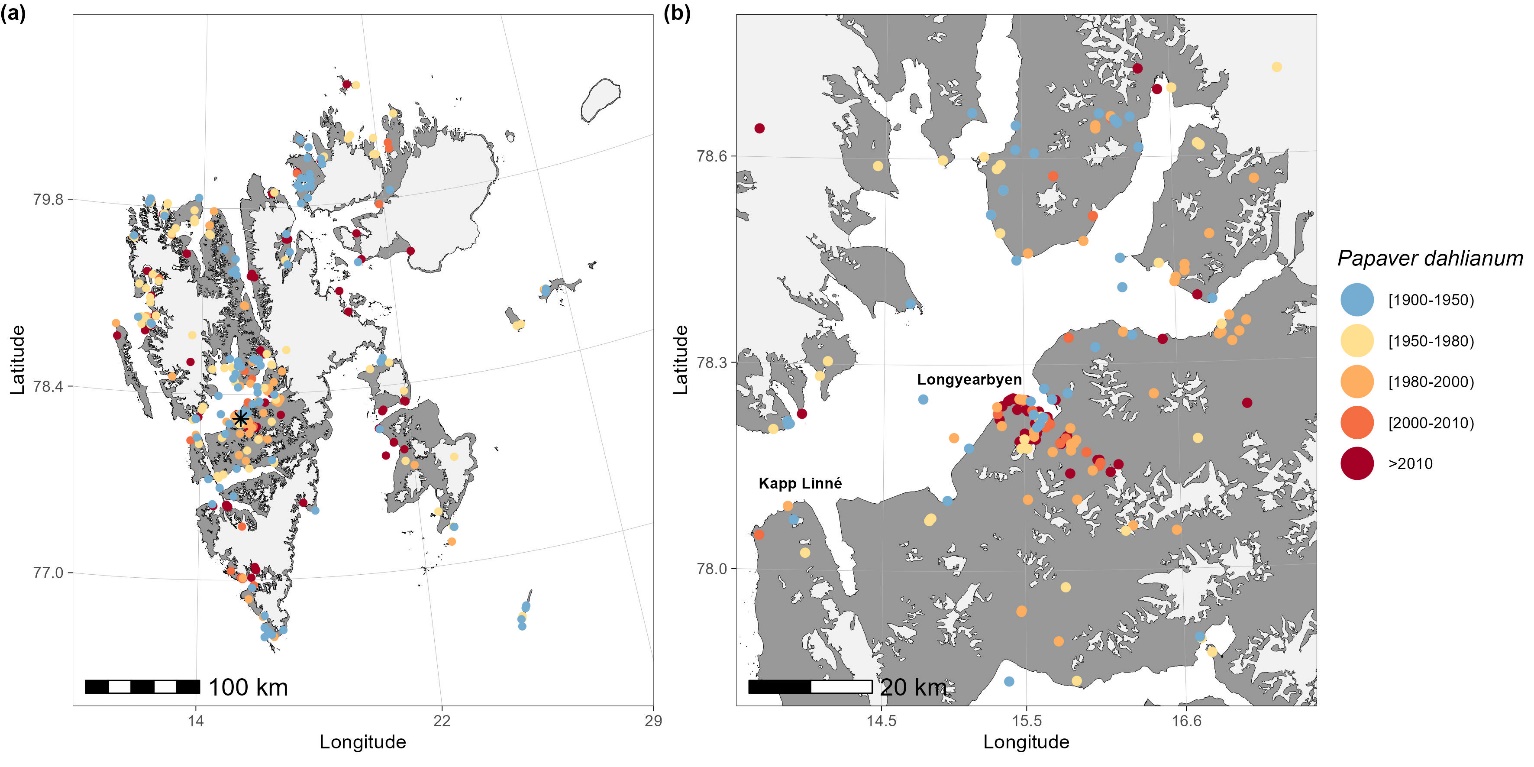


**Figure S1.1**: Historical occurrence records of *Papaver dahlianum* at **(a)** Svalbard (Longyearbyen indicated by a star-shaped symbol) and **(b)** Isfjorden from 1900 until now. Warmer colours indicate more recent years. Coordinate positions were derived from the Global Biodiversity Information Facility (GBIF) database using the R package ‘rgbif’ (Chamberlain et al., 2024). Maps were plotted using the R package ‘PlotSvalbard’ (Vihtakari, 2020). Old records of species occurrence may present spatial inaccuracies.


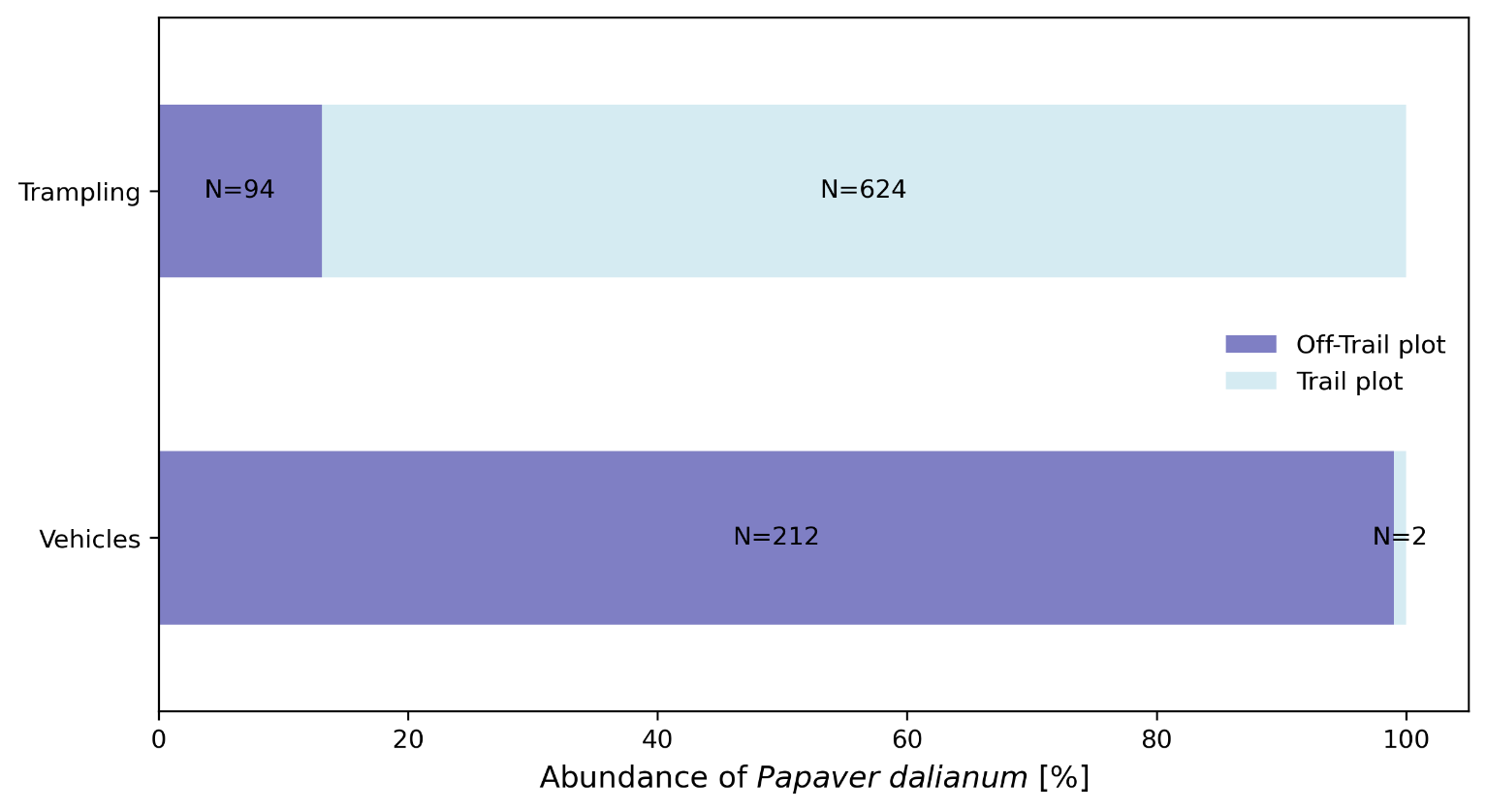


**Figure S1.2**: Individual abundance (%) of *Papaver dahlianum* on the main trail plots (light blue) and all perpendicular off-trail plots (dark blue) for different levels of trail disturbance. “Vehicles” = disturbance from vehicles across the initial tractor track close to Isfjord Radio station. “Trampling” = hikers (and animal) trampling on the remaining hiking trail. Numbers of individuals (N) are reported on the bars. Individual abundance was observed according to the 2023 ground survey (see Sect. 2.2 for more details).


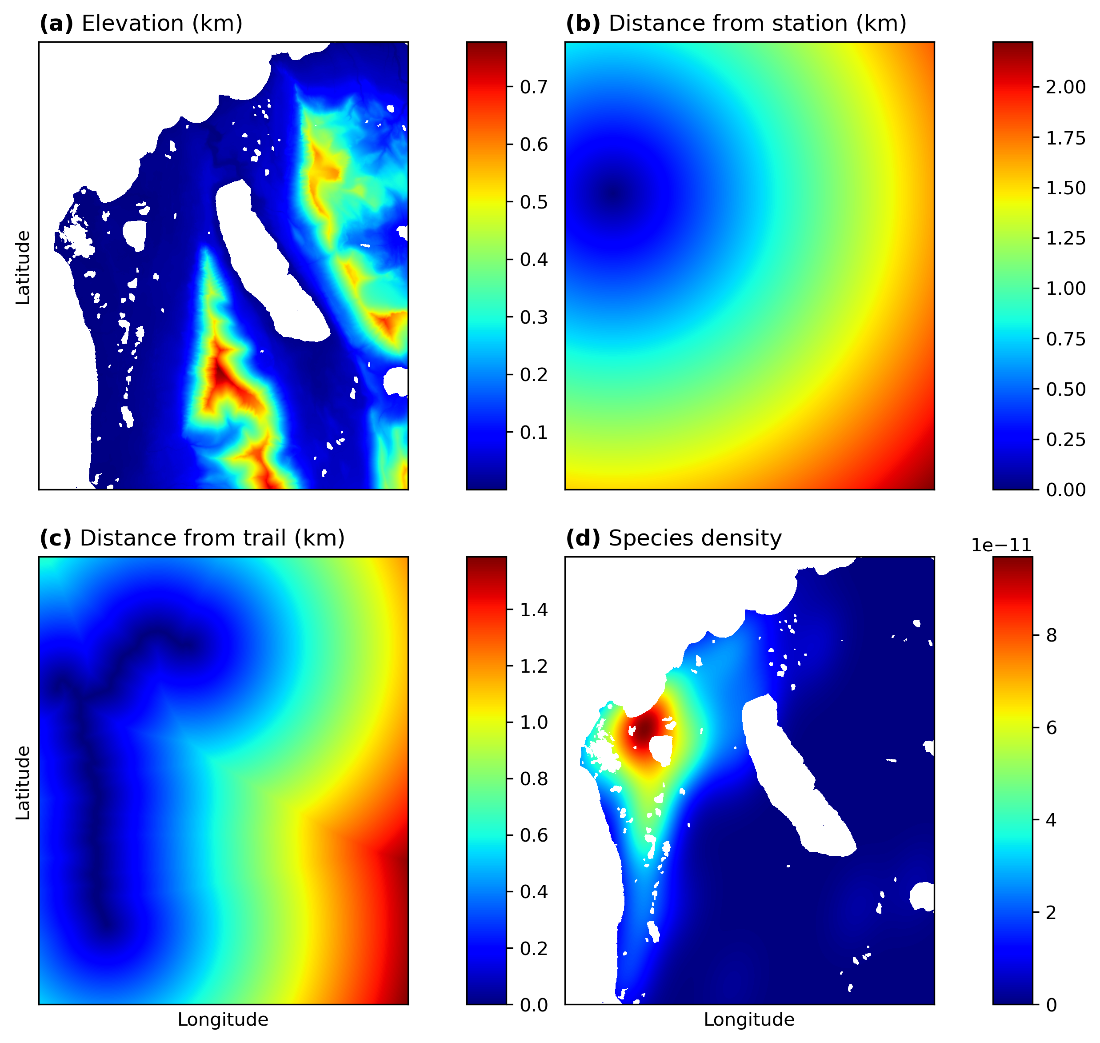


**Figure S1.3**: Predictors (a-c) for the density of *Papaver dahlianum* (d) of the GLM. (d) The normalized density of *Papaver dahlianum* was calculated with a Kernel Density Estimation (KDE) from historical records (1994, 2012, 2018).


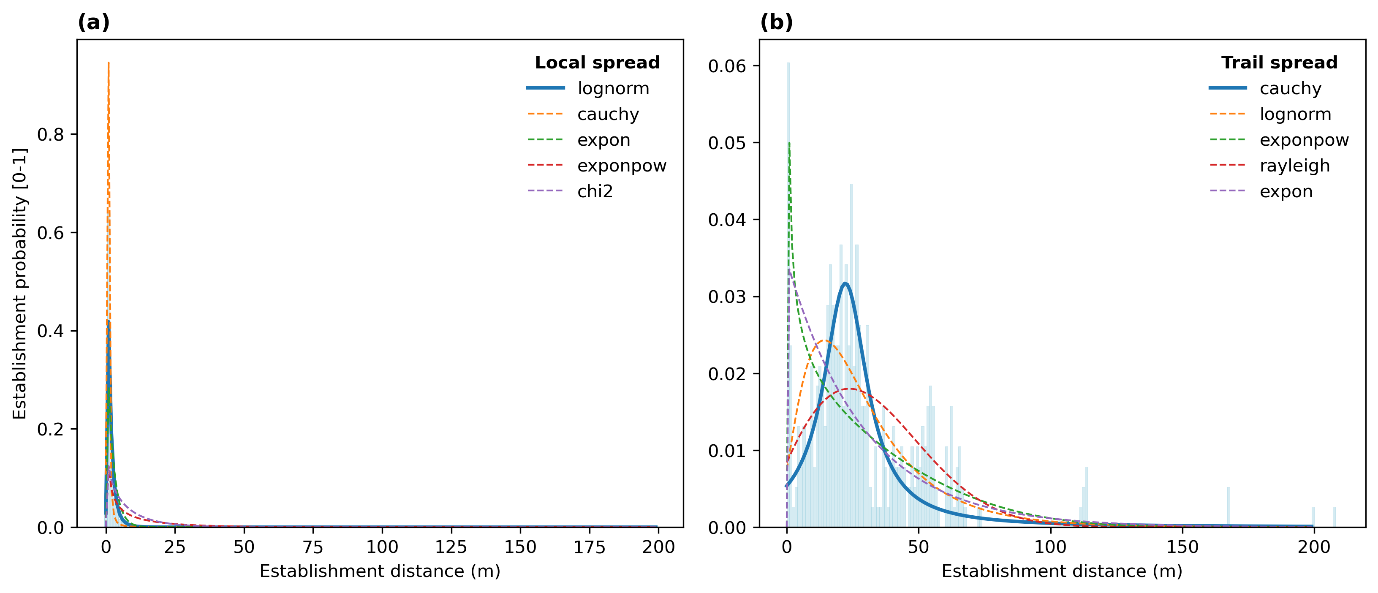


**Figure S1.4**: Top-5 best kernel fits from contemporary *Papaver dahlianum* abundance along the main hiking trail starting from Isfjord Radio station. The solid blue lines indicate the best-fitted kernel. The light blue bars indicate the density of observed establishment distances from the source. Kernels were fitted under the assumption of either (a) local spread (source is the closest individual) or (b) spread from the trail (source is the closest individual on the trail). For Probability Density Functions (PDFs) and best parameters, see Table 2.


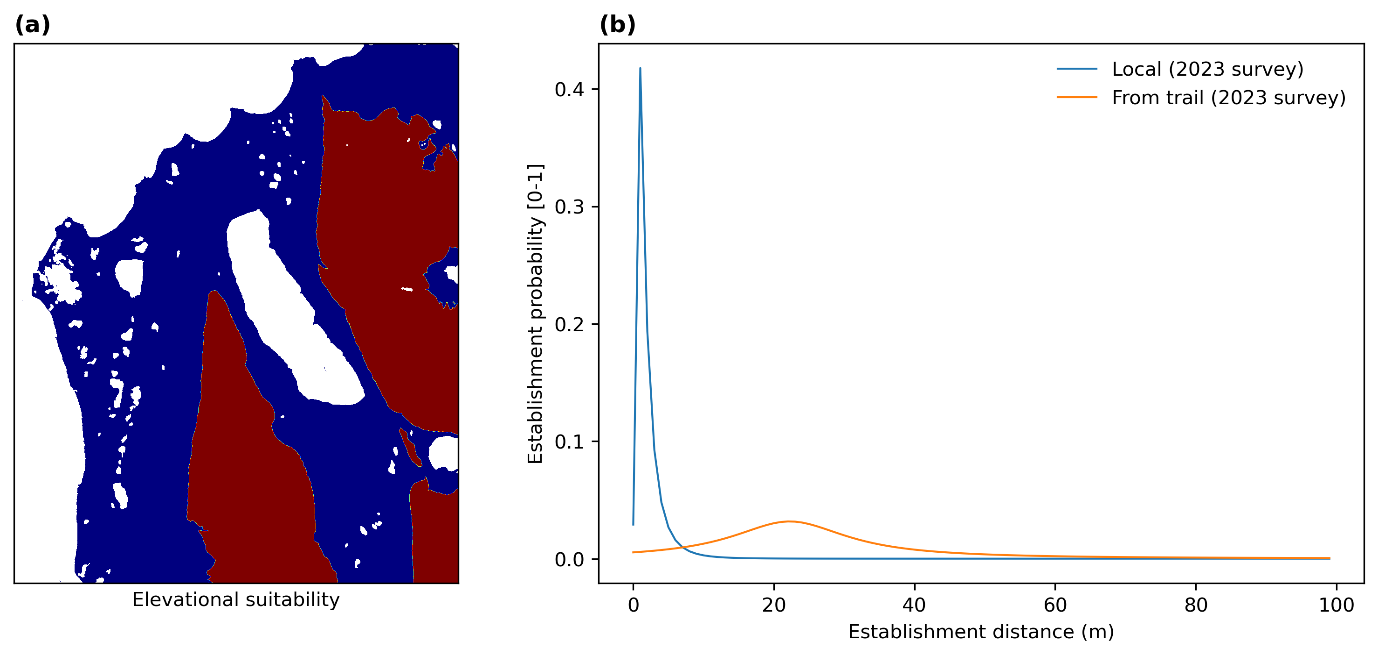


**Figure S1.5**: (a) Topographic suitability for establishment (0 = red, 1 = blue) based on elevation as fitted by the generalized linear model GLM(Species density $\boldsymbol{\sim}$ elevation) from historical records (1994, 2012, 2018). (b) Best kernel fits from contemporary *Papaver dahlianum* abundance along the main hiking trail starting from Isfjord Radio station. Kernels were fitted under the assumption of either local spread (blue) or spread from the trail (orange). For Probability Density Functions (PDFs) and best parameters (and visual representation), see Table 2 (Fig. S1.4).

**References**

Chamberlain, S., Barve, V., Mcglinn, D., Oldoni, D., Desmet, P., Geffert, L., & Karthik Ram. (2024). rgbif: Interface to the Global Biodiversity Information Facility API. <https://CRAN.R-project.org/package=rgbif>

Vacca, G. (2020). WEB open drone map (WebODM) a software open source to photogrammetry process. In Fig Working Week 2020. *Smart surveyors for land and water management*. <https://github.com/OpenDroneMap/WebODM>

Vihtakari, M. (2020). PlotSvalbard: PlotSvalbard - Plot research data from Svalbard on maps. <https://github.com/MikkoVihtakari/PlotSvalbard>
